# Clinical Complete Response and Organ Preservation Strategies in Rectal Cancer: A Real-World Single-Center Experience Clinical Complete Response and Organ Preservation in Rectal Cancer

**DOI:** 10.3390/cancers18050763

**Published:** 2026-02-27

**Authors:** J. A. Encarnación, N. Ibáñez, I. De la Fuente, P. Ruiz, S. González, B. Quiles, M. Sánchez, Y. Bautista, C. Rodríguez, J. A. Nadal, M. Marín, G. Marín-Zafra, M. Guirao, Q. Hernández, J. Abrisqueta, I. Abellán, M. Montoya, A. Ono, G. Carbonell, L. Frutos, E. Ortiz, C. Manso, M. Royo-Villanova, J. L. Alonso-Romero

**Affiliations:** 1 Servicio de Oncología Radioterápica, University of Murcia, 30100 Murcia, Spain; isabeldelafuente123@gmail.com (I.D.l.F.); dm.oncoarrixaca2@gmail.com (P.R.); sandra.19792@gmail.com (S.G.); beaquihe@gmail.com (B.Q.); sanchez.perez.marta@gmail.com (M.S.); docyasminabm@gmail.com (Y.B.); cristina.rodriguez.mnez@gmail.com (C.R.); josenadalgarcia42@gmail.com (J.A.N.); miguelmarin75@hotmail.com (M.M.); gemarza@gmail.com (G.M.-Z.); marta.guirao@carm.es (M.G.); josel.alonso2@carm.es (J.L.A.-R.); 2 Department of Radiation Oncology, Virgen De La Arrixaca University Clinical Hospital, Carretera Madrid-Cartagena s/n, 30120 Murcia, Spain; 3 Murcian Institute for Biomedical Research “Pascual Parrilla” (IMIB), Carretera Madrid-Cartagena s/n, 30120 Murcia, Spain; noelia.ibc@hotmail.es; 4 Department of General Surgery, Virgen De La Arrixaca University Clinical Hospital, 30120 Murcia, Spain; quihera@gmail.com (Q.H.); j_abris@hotmail.com (J.A.); israelabellanmorcillo@gmail.com (I.A.); mjmontoya@yahoo.es (M.M.); 5 Department of Medical Oncology, Virgen De La Arrixaca University Clinical Hospital, 30120 Murcia, Spain; 6 Department of Gastroenterology, Virgen De La Arrixaca University Clinical Hospital, 30120 Murcia, Spain; ono.akiko@gmail.com; 7 Department of Radiodiagnostic, Virgen De La Arrixaca University Clinical Hospital, 30120 Murcia, Spain; guillermo.carbonell@carm.es; 8 Department of Nuclear Medicine, Virgen De La Arrixaca University Clinical Hospital, 30120 Murcia, Spain; laura.frutos@yahoo.es; 9 Department of Pathological Anatomy, Virgen De La Arrixaca University Clinical Hospital, 30120 Murcia, Spain; edu_or@yahoo.es; 10 Department of Intensive Care Medicine, Virgen De La Arrixaca University Clinical Hospital, 30120 Murcia, Spain; clara92mm@hotmail.com (C.M.); mariorvr@hotmail.com (M.R.-V.)

**Keywords:** rectal cancer, clinical complete response, watch and wait, neoadjuvant therapy, organ preservation, radiotherapy, total neoadjuvant therapy, real-world evidence

## Abstract

Background: The management of rectal cancer has evolved toward response-adapted strategies, including organ preservation in selected patients achieving a clinical complete response (cCR) after neoadjuvant treatment. However, most available evidence derives from clinical trials, and data from real-world clinical practice remain limited. Methods: We conducted a retrospective observational cohort study including consecutive patients with rectal adenocarcinoma treated at a tertiary referral center between January 2021 and December 2025. Baseline clinical, tumor-related, and treatment characteristics were collected. Tumor response was assessed using clinical, endoscopic, and radiological criteria. The primary endpoint was the rate of clinical complete response and the implementation of watch-and-wait strategies. Secondary endpoints included recurrence patterns and exploratory oncologic outcomes according to baseline tumor characteristics. Results: A total of 229 patients were identified, of whom 148 were evaluable for treatment response. Clinical complete response was documented in 56 patients (37.8%), and a watch-and-wait strategy was implemented in 42 patients (28.4%). Higher cCR rates were observed in patients with stage I–II disease and in tumors measuring < 4 cm on baseline magnetic resonance imaging, with cCR rates exceeding 55% in this subgroup. Tumors ≥ 4 cm showed substantially lower response rates. Clinical complete responses were observed across both short-course radiotherapy plus chemotherapy and long-course chemoradiotherapy regimens in patients with small tumors and early-stage disease. Tumor distance from the anal verge was not consistently associated with response. With a median follow-up of 26 months in the watch-and-wait group, five recurrences were observed, including three local recurrences. Conclusions: In this real-world cohort, baseline tumor size and clinical stage were the main determinants of clinical complete response and eligibility for organ-preservation strategies in rectal cancer. Small tumors (<4 cm) showed high response rates regardless of neoadjuvant regimen. These findings support response-adapted, individualized treatment strategies and highlight the importance of tumor burden in selecting candidates for non-operative management in routine clinical practice.

## Simple Summary

Rectal cancer treatment is increasingly tailored according to how well tumors respond to preoperative therapy. In selected patients who achieve a clinical complete response after neoadjuvant treatment, surgery may be safely avoided using a “watch-and-wait” strategy, thereby preserving the rectum and reducing long-term functional impairment. However, most available evidence comes from clinical trials, and data from routine clinical practice remain limited. In this real-world single-center study, we evaluated treatment response and the implementation of organ-preservation strategies in 148 patients with rectal cancer. Clinical complete response was observed in 37.8% of evaluable patients, and a watch-and-wait approach was adopted in 28.4%. Baseline tumor size and clinical stage were the main determinants of response. Tumors smaller than 4 cm showed particularly high response rates, regardless of the neoadjuvant regimen used. These findings support individualized, response-adapted treatment strategies and highlight the importance of tumor burden in selecting candidates for non-operative management in everyday clinical practice.

## 1. Introduction

Rectal cancer accounts for approximately one-third of all colorectal cancers and remains a relevant clinical challenge due to its anatomical, biological, and therapeutic complexity [1]. Over recent decades, the management of locally advanced rectal cancer has undergone substantial evolution, driven by improvements in pelvic magnetic resonance imaging–based staging, refinements in surgical techniques, and optimization of multimodal treatment strategies [2,3].

Traditionally, the standard treatment for locally advanced tumors has consisted of neoadjuvant chemoradiotherapy followed by surgery with total mesorectal excision and, in selected cases, adjuvant chemotherapy [4]. However, this approach is associated with significant morbidity, including bowel, urinary, and sexual dysfunction, as well as a relevant negative impact on quality of life, particularly in tumors of the mid and lower rectum [5].

In this context, new therapeutic strategies have emerged with the aim of improving systemic disease control and, potentially, avoiding surgery in selected subgroups of patients. Intensification of neoadjuvant treatment through total neoadjuvant therapy (TNT), as well as the adoption of sequential chemotherapy and radiotherapy regimens, has been shown to increase tumor response rates, including pathological and clinical complete response [6,7,8]. These advances have renewed interest in organ-preservation strategies, such as the “watch and wait” approach, in patients achieving a documented clinical complete response after neoadjuvant treatment [9,10,11].

Nevertheless, the available evidence largely derives from clinical trials with strict selection criteria, which limits its generalizability to routine clinical practice [12]. In real-world settings, patients exhibit considerable heterogeneity in terms of tumor stage, anatomical characteristics, comorbidities, and treatment regimens, complicating the interpretation of outcomes and the identification of subgroups that may benefit from specific strategies [13].

In this scenario, contemporary observational studies are of particular value, as they allow evaluation of the implementation of different therapeutic strategies in real-world healthcare settings and the analysis of oncologic outcomes and management patterns outside the context of clinical trials [14,15]. The aim of the present study is to describe the clinical, tumor-related, and therapeutic characteristics of patients with rectal cancer treated in a real-world clinical practice setting, as well as to analyze oncologic outcomes with particular emphasis on tumor response and the application of organ-preservation strategies.

## 2. Materials and Methods

### 2.1. Study Design and Patient Selection

We conducted a retrospective observational cohort study including consecutive patients diagnosed with rectal adenocarcinoma and treated at the Hospital Clínico Universitario Virgen de la Arrixaca (HCUVA), Murcia, Spain, between January 2021 and the date of data cutoff. The study was approved by the local Institutional Review Board and conducted in accordance with the Declaration of Helsinki. Given the retrospective and non-interventional nature of the study, the requirement for informed consent was waived.

Eligible patients were adults (≥18 years) with histologically confirmed rectal adenocarcinoma, defined as tumors located within 15 cm from the anal verge as assessed by colonoscopy and/or pelvic magnetic resonance imaging (MRI). Patients were included if they received any oncologic treatment with curative or disease-control intent at our institution. Patients with incomplete baseline data or without follow-up information were excluded from outcome analyses.

### 2.2. Staging and Baseline Assessment

Baseline staging was performed according to standard clinical practice and included digital rectal examination, colonoscopy with biopsy, pelvic MRI, and thoracoabdominal computed tomography (CT). Tumors were staged using the TNM classification system applicable at the time of diagnosis. Clinical stage was recorded based on imaging findings prior to any oncologic treatment.

Tumor size was preferentially derived from baseline pelvic MRI. When MRI measurements were unavailable, tumor length reported at colonoscopy was used as an alternative estimate. For analytical purposes, tumor size was additionally categorized using cut-off values of 3 cm and 4 cm, based on previously published evidence suggesting their prognostic and predictive relevance in rectal cancer treated with neoadjuvant strategies [16]. Tumor distance from the anal verge was recorded based on MRI or colonoscopy reports. Additional baseline variables collected included age at diagnosis, sex, clinical T and N stage, overall clinical stage, and year of diagnosis.

### 2.3. Treatment Strategies

Treatment decisions were made within a multidisciplinary tumor board and reflected routine clinical practice during the study period. Patients received heterogeneous treatment approaches according to tumor stage, anatomical characteristics, patient-related factors, and evolving institutional protocols.

Neoadjuvant treatment strategies included long-course chemoradiotherapy, short-course radiotherapy with delayed surgery, total neoadjuvant therapy, or chemotherapy alone in selected cases. Surgical management consisted of total mesorectal excision when indicated. Adjuvant chemotherapy was administered at the discretion of the treating medical oncologist.

Treatment allocation was decided by a multidisciplinary tumor board. In general, neoadjuvant treatment was recommended for stage II–III rectal cancer and for selected stage I tumors when organ-preservation was pursued (e.g., low-rectal tumors where maximal response could increase the likelihood of non-operative management) or when baseline MRI suggested higher-risk features. For stage IV cases included in this cohort, all patients had oligometastatic disease with curative-intent strategies, and were managed with combined systemic therapy and local treatment (rectal and metastatic sites) according to multidisciplinary assessment.

In patients achieving a documented clinical complete response after neoadjuvant treatment, a non-operative management strategy (“watch and wait”) could be adopted. Clinical complete response was defined as the absence of residual tumor on endoscopic assessment and imaging studies, according to institutional criteria consistent with the International Watch & Wait Consensus. The decision to proceed with a watch-and-wait approach was made by the multidisciplinary team and documented in the medical record.

### 2.4. Watch-And-Wait Strategy/Response Assessment

The decision to adopt a watch-and-wait strategy was restricted to patients with a documented clinical complete response at restaging, defined by the absence of residual disease on both endoscopic evaluation and pelvic MRI. Patients in whom restaging imaging or endoscopy suggested persistent or equivocal residual disease were referred to surgery according to standard practice. In some cases, a complete response was only confirmed on the surgical specimen, representing pathological rather than preoperative clinical complete responses; these patients were not eligible for non-operative management.

Clinical complete response was assessed at restaging using a combination of digital rectal examination, endoscopic evaluation, and pelvic magnetic resonance imaging (MRI), in accordance with institutional practice and consistent with the International Watch & Wait Consensus. Endoscopic criteria for cCR included the absence of residual tumor, with findings limited to a flat white scar, telangiectasia, or mucosal whitening, without ulceration or nodularity. On MRI, cCR was defined by the absence of residual intermediate-signal tumor on T2-weighted images, with replacement by fibrosis or low-signal intensity, no suspicious lymph nodes, and no evidence of extramural disease progression.

Response assessment was typically performed 8–12 weeks after completion of neoadjuvant treatment, with timing adapted according to treatment sequence (short-course radiotherapy, long-course chemoradiotherapy, or total neoadjuvant therapy) and multidisciplinary evaluation.

### 2.5. Data Collection and Follow-Up

Clinical, radiological, pathological, and treatment-related data were extracted retrospectively from electronic medical records. Follow-up was conducted according to institutional protocols and included regular clinical evaluation, endoscopic assessment, imaging studies, and laboratory tests.

Pathological response was assessed in resected specimens according to standard histopathological criteria, including ypT and ypN stage and tumor regression grade when available. For patients managed with watch and wait, response was assessed using serial clinical, endoscopic, and radiological evaluations.

Recurrence was defined as radiologically or histologically confirmed local or distant disease after completion of primary treatment. Date and pattern of recurrence (local, distant, or combined) were recorded. Overall survival was defined as the time from diagnosis to death from any cause or last follow-up.

### 2.6. Study Endpoints

The primary endpoint of the study was treatment response, particularly the rate of clinical complete response and the adoption of watch-and-wait strategies in a real-world setting. Secondary endpoints included recurrence patterns and exploratory oncologic outcomes according to baseline tumor characteristics, including clinical stage and tumor size.

### 2.7. Statistical Analysis

Descriptive statistics were used to summarize baseline characteristics and treatment details. Categorical variables were expressed as absolute numbers and percentages, while continuous variables were reported as medians and ranges or interquartile ranges, as appropriate.

Statistical analyses focused on descriptive and exploratory assessments of treatment response and clinical outcomes. Analyses were performed using standard statistical software.

No formal comparative, hypothesis-testing, or multivariable analyses were performed. Observations regarding treatment regimens should therefore be interpreted as descriptive and exploratory, without implying statistical equivalence or superiority.

### 2.8. Data Availability

The data supporting the findings of this study are available from the corresponding author upon reasonable request. Data are not publicly available due to privacy and ethical restrictions.

## 3. Results

### 3.1. Patient Characteristics

**Figure 1:**
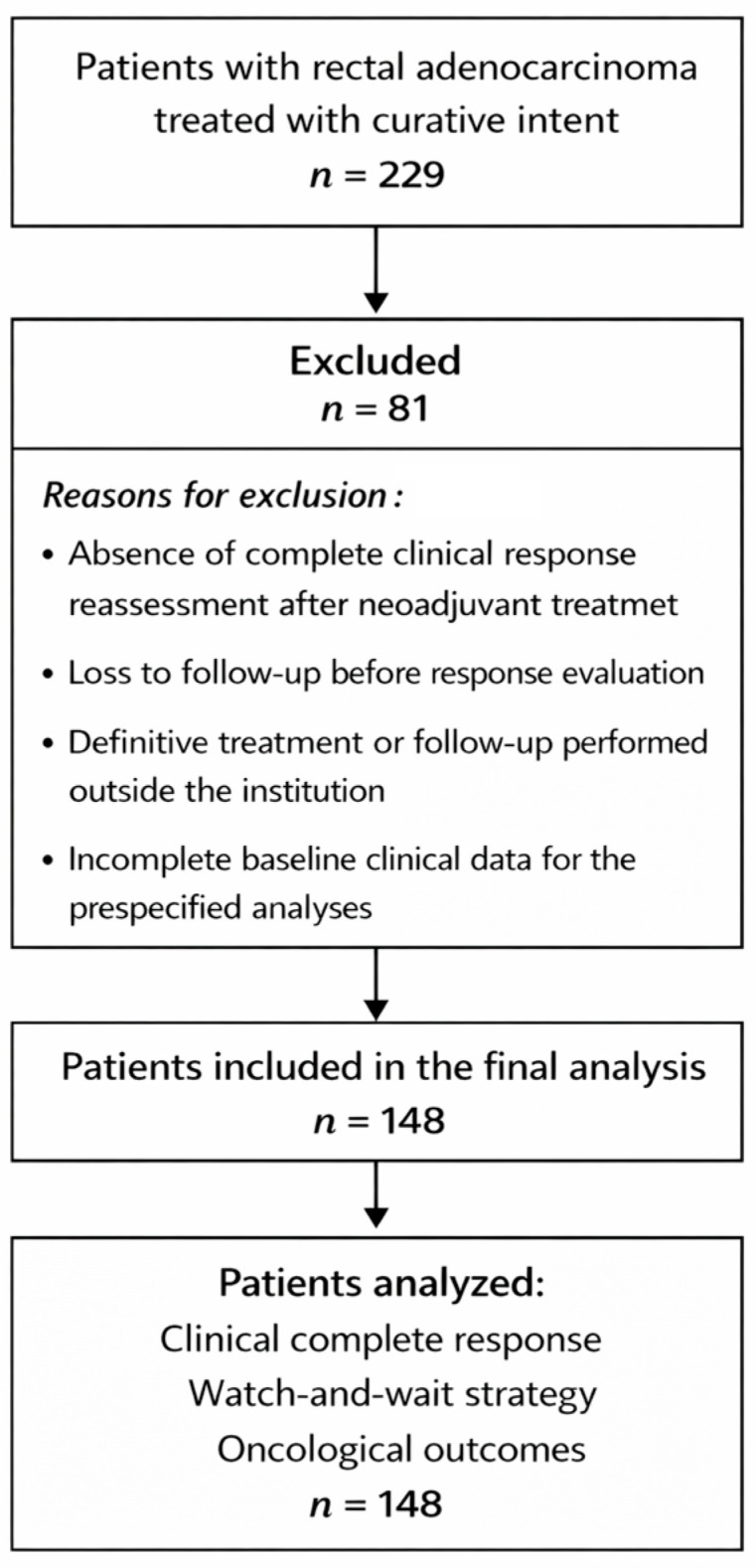
Flow chart of patient selection.

A total of 229 consecutive patients diagnosed with rectal adenocarcinoma and treated at the Hospital Clínico Universitario Virgen de la Arrixaca between 2021 and the date of data cutoff (December 2025) were identified (Figure 1). Response assessment was available in 148 patients, who constituted the response-evaluable population for analyses related to treatment response.

Patients not included in the response-evaluable population were excluded due to incomplete restaging data, absence of standardized response assessment, early postoperative referral, or loss to follow-up before response evaluation. These exclusions were mainly related to logistical or data-availability issues rather than baseline oncologic characteristics. Importantly, the excluded patients included a heterogeneous mix of clinical stages and treatment intents, and no systematic exclusion based on tumor stage or response likelihood was applied.

Baseline clinical, tumor-related, and treatment characteristics of the response-evaluable population are summarized in Table 1.

### 3.2. Treatment Strategies

Patients received heterogeneous therapeutic approaches according to tumor stage, anatomical characteristics, and the evolution of institutional protocols (Table 2). During the period 2021–2023, the predominant strategy was short-course radiotherapy (5 Gy × 5 fractions) combined with chemotherapy, which represented the preferred regimen during that time interval. Subsequently, long-course chemoradiotherapy regimens (50 Gy with capecitabine) and combined approaches incorporating systemic chemotherapy were more frequently adopted according to tumor stage.

Surgical resection with total mesorectal excision was performed when indicated. A subgroup of patients who achieved a documented clinical complete response after neoadjuvant treatment was managed using a non-operative watch-and-wait strategy following multidisciplinary evaluation.

### 3.3. Clinical Complete Response

Among the 148 response-evaluable patients, a clinical complete response (cCR) was documented in 56 patients, corresponding to 37.8% of the evaluable cohort. A watch-and-wait strategy was implemented in 42 patients (28.4%). Not all patients achieving complete response were managed with a watch-and-wait strategy, as eligibility required confirmation of clinical complete response at preoperative restaging. In a subset of patients, complete response was only documented on the surgical specimen following surgery that had been indicated due to suspicious findings on MRI and/or endoscopy.

### 3.4. Clinical Complete Response According to Baseline Clinical Stage

Rates of cCR varied according to baseline clinical stage. The highest rates were observed in patients with stage I and II disease, with a progressive decrease as stage advanced (Table 3).

### 3.5. Tumor Size and Clinical Complete Response

Baseline tumor size emerged as the most relevant factor associated with achievement of cCR. Patients with tumors measuring <4 cm showed substantially higher cCR rates compared with those with larger tumors (Table 4 and Table 5).

Notably, cCR rates decreased markedly in tumors ≥ 4 cm, and only sporadic responses were observed in tumors larger than 6 cm, reflecting an inverse relationship between baseline tumor size and the likelihood of complete response (Table 4 and Table 5).

### 3.6. Watch-And-Wait Strategy

**Figure 2:**
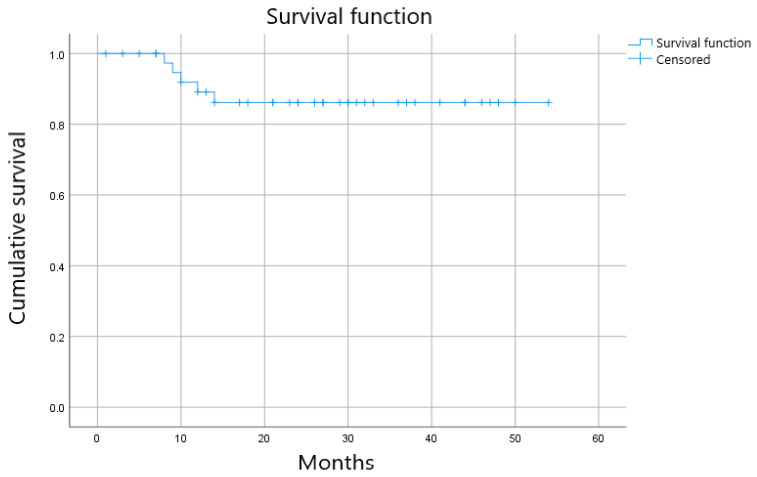
Kaplan–Meier estimate of local regrowth–free probability in patients managed with a watch-and-wait strategy. Tick marks indicate censored observations. Patients without recurrence were censored at last follow-up (median follow-up: 26 months; range: 1–54 months).

A watch-and-wait strategy was implemented in 42 patients (28.4% of evaluable cases). Not all patients achieving cCR were managed non-operatively, reflecting additional clinical selection criteria beyond tumor response alone. Among patients managed with a watch-and-wait approach, the median follow-up was 26 months (range, 1–54 months) (Figure 2).

Among the 42 patients for whom a watch-and-wait strategy was implemented, a total of five recurrences were observed during follow-up. Three were local recurrences (corresponding to two T4 tumors and one T2 tumor), and two were distant recurrences. Local recurrences occurred at 8, 9, and 12 months after response assessment, while distant recurrences were detected at 10 and 14 months. All local recurrences occurred in patients managed with a watch-and-wait strategy and were detected during scheduled surveillance. Salvage surgery was performed in all cases, achieving local disease control.

### 3.7. Interaction Between Tumor Size, Stage, and Treatment Strategy

When focusing on patients with tumors < 4 cm, cCRs were observed across different neoadjuvant regimens. These findings are descriptive and exploratory, and no formal comparative analyses were performed. In this subgroup, patients treated with short-course radiotherapy (5 × 5 Gy) plus chemotherapy achieved 16 clinical complete responses, while 10 patients did not achieve cCR. Patients treated with long-course chemoradiotherapy (50 Gy with capecitabine) achieved 15 cCRs, with 9 patients not achieving cCR.

Similarly, among patients with stage I–II disease, those treated with short-course radiotherapy plus chemotherapy achieved 10 cCRs and 10 non-cCRs, whereas patients treated with long-course chemoradiotherapy achieved 12 cCRs compared with 13 patients without cCR.

### 3.8. Tumor Distance from the Anal Verge

Tumor distance from the anal verge did not show a consistent association with either cCR or the adoption of watch-and-wait strategies (Table 6). Response rates were similar across distance categories, suggesting that anatomical location alone was not a decisive factor for response in this cohort. Overall, these findings indicate that tumor distance from the anal verge was not a relevant determinant of response in this cohort.

## 4. Discussion

In this real-world, single-center cohort, we evaluated treatment patterns, tumor response, and organ-preservation strategies in patients with rectal cancer managed in routine clinical practice over a contemporary period. Several relevant findings emerge from our analysis, particularly regarding the role of baseline tumor characteristics in predicting response and the evolution of neoadjuvant treatment strategies over time.

The overall rate of clinical complete response (cCR) in our response-evaluable population was 37.8%, with a watch-and-wait strategy implemented in 28.4% of patients. These rates are consistent with those reported in contemporary series incorporating intensified neoadjuvant approaches and organ-preservation strategies [9,10,11,15]. Importantly, not all patients achieving cCR were managed non-operatively, highlighting that real-world clinical decision-making extends beyond response assessment alone and incorporates tumor stage, patient-related factors, and perceived oncologic risk.

Baseline tumor size emerged as the most relevant determinant of treatment response in our cohort. Patients with tumors measuring less than 4 cm achieved substantially higher rates of clinical complete response and were more frequently managed with watch-and-wait strategies. In contrast, response rates declined markedly in tumors ≥ 4 cm, with only sporadic responses observed in tumors larger than 6 cm. These findings reinforce previous observations indicating that baseline tumor burden is a critical factor influencing response and eligibility for organ-preservation strategies, potentially outweighing treatment-related variables [10,11,14].

Tumor measurements were primarily derived from baseline pelvic MRI, which represents the standard modality for local staging in rectal cancer. When MRI measurements were unavailable, colonoscopic tumor size was used as an alternative, reflecting routine clinical practice. Although some degree of measurement variability between modalities cannot be excluded, both MRI and endoscopic tumor length are commonly used in real-world settings to estimate tumor burden.

The cut-off values used in this study (<3 cm, 3–4 cm, and ≥4 cm) were selected a priori based on previously published evidence suggesting that smaller tumor size is associated with higher rates of complete response and suitability for organ-preservation strategies. In particular, thresholds around 3–4 cm have been reported as clinically meaningful in response-adapted approaches and were supported by the marked decrease in response rates observed beyond this size in our cohort.

Patients without MRI-based measurements were not excluded from analysis in order to preserve the real-world nature of the cohort; instead, alternative size estimates from colonoscopy were used. While this approach may introduce some imprecision, it avoids systematic exclusion of patients and reflects routine clinical decision-making.

Clinical stage also influenced response rates, with the highest cCR rates observed in patients with stage I–II disease and a progressive decrease with advancing stage. Although occasional cCRs were observed even in stage IV disease, the adoption of watch-and-wait strategies in advanced stages was uncommon. This cautious approach reflects ongoing concerns regarding oncologic safety and is consistent with current guideline recommendations and real-world practice patterns [12,13].

An additional observation of interest in our cohort relates to the pattern of local recurrence among patients managed with a watch-and-wait strategy. Although the overall number of recurrences was limited, local regrowth occurred predominantly in patients with initially advanced primary tumors, including cases staged as T4 at baseline. This finding suggests that baseline local tumor extension may influence the risk of local failure following non-operative management, even in the presence of an initial clinical complete response. Importantly, all local recurrences in this subgroup were detected during structured follow-up and were successfully managed with salvage surgery, allowing definitive pathological assessment and local disease control.

An important observation of our study relates to the evolution of neoadjuvant treatment strategies during the study period. During the earlier years (2021–2023), short-course radiotherapy combined with chemotherapy (5 × 5 Gy + QT) was the predominant approach at our institution and was frequently applied even in patients with early-stage disease and favorable tumor characteristics. In retrospect, this strategy may have represented overtreatment in selected low-risk patients. Despite this, outcomes in patients with stage I–II disease and small tumors treated with 5 × 5 Gy plus chemotherapy were comparable to those observed in more recent patients treated with long-course chemoradiotherapy (50 Gy with capecitabine), with similar rates of clinical complete response across both groups.

These findings are in line with accumulating evidence suggesting that long-course chemoradiotherapy may be superior to short-course radiotherapy when maximal tumor regression and response optimization are key objectives. Meta-analyses and randomized studies have shown higher rates of pathological and clinical complete response with long-course chemoradiotherapy compared with short-course regimens, particularly in tumors of the mid and lower rectum, thereby facilitating organ-preservation strategies [17,18,19]. Current clinical practice guidelines also favor long-course chemoradiotherapy in scenarios where tumor downsizing and response assessment are central to treatment decision-making [20].

Beyond systemic treatment intensification, radiation dose escalation to the primary tumor represents a promising strategy to further increase rates of clinical complete response and expand eligibility for organ-preservation approaches. In particular, the use of contact X-ray brachytherapy (CXB, Papillon technique) as a boost following external beam radiotherapy has consistently demonstrated improved local tumor control, higher rates of complete clinical response, and increased sphincter preservation in selected patients with early and intermediate rectal cancer [21,22,23,24,25]. Long-term data from the Lyon R96-02 randomized trial and subsequent updates have shown durable oncologic outcomes with this approach, while more recent prospective studies, including the OPERA trial, have confirmed the potential of CXB boost to enhance organ preservation without compromising local control [20,21,22,23]. Contemporary reviews further support the role of CXB as an effective and safe dose-escalation strategy when delivered in experienced centers [25,26].

In parallel, dose escalation using external beam radiotherapy techniques, such as intensity-modulated radiotherapy (IMRT), volumetric modulated arc therapy (VMAT), or simultaneous integrated boost (SIB), has also been explored as a means to increase tumor response. The RECTAL-BOOST trial demonstrated that radiotherapy dose escalation to the primary tumor can increase complete response rates, although at the cost of increased acute toxicity in some cases [27]. Recent reviews highlight the heterogeneity of boost techniques and doses but suggest that EBRT-based dose escalation may represent an alternative strategy in centers without access to CXB, particularly when carefully selected and delivered with modern radiation techniques [28].

In our cohort, tumor distance from the anal verge did not show a consistent association with response or the adoption of watch-and-wait strategies. This suggests that anatomical location alone should not preclude consideration of response-based management when other favorable tumor characteristics are present, supporting a more individualized approach to patient selection [14].

Beyond clinical and radiological factors, emerging molecular and biological predictors of response are expected to play an increasingly important role in personalizing treatment strategies for rectal cancer and in refining patient selection for organ-preservation approaches. In particular, mismatch repair deficiency and microsatellite instability–high (dMMR/MSI-H) status, as well as specific genomic alterations and transcriptomic or radiomic signatures, have been associated with distinct response patterns to neoadjuvant therapies and immunotherapy-based strategies in selected populations [29,30].

In the present real-world cohort, molecular biomarkers were not systematically available due to the retrospective design and the evolving implementation of routine molecular profiling during the study period. As a result, our analyses focused on readily available clinical, anatomical, and radiological variables that continue to guide treatment decisions in everyday practice. Our findings should therefore be interpreted within this clinically driven framework and viewed as complementary to, rather than competitive with, emerging biomarker-based approaches.

Similarly, although recurrence patterns and early oncologic outcomes are reported, the relatively limited follow-up duration precludes definitive conclusions regarding long-term oncologic safety and survivorship. Accordingly, our results should be considered hypothesis-generating and underscore the need for future prospective studies integrating clinical, radiological, and molecular data with prolonged follow-up to optimize response-adapted and organ-preserving strategies.

The strengths of this study include its real-world design, inclusion of consecutive patients, and reflection of routine multidisciplinary decision-making across a period of evolving treatment paradigms. However, several limitations should be acknowledged. The retrospective, single-center nature of the study limits generalizability, and follow-up duration may be insufficient to fully capture long-term oncologic outcomes, particularly in patients managed with watch and wait. In addition, treatment heterogeneity and missing data for some baseline variables reflect routine clinical practice and may introduce bias. Because response analyses were restricted to patients with complete post-treatment restaging, selection bias cannot be excluded. In particular, patients excluded from the response-evaluable cohort may have differed in baseline stage distribution and/or treatment intent, which could have influenced the observed cCR rate.

## 5. Conclusions

In conclusion, our findings underscore the central role of baseline tumor size and stage in determining response and eligibility for organ-preservation strategies in rectal cancer. While short-course radiotherapy combined with chemotherapy was frequently used in earlier years and may have resulted in overtreatment of selected low-risk patients, comparable outcomes were observed with long-course chemoradiotherapy in similar subgroups. Emerging evidence suggests that radiotherapy dose escalation, particularly through contact X-ray brachytherapy or selected EBRT boost strategies, may further increase complete response rates and broaden the population eligible for non-operative management, warranting prospective evaluation and careful patient selection.

## Figures and Tables

**Table 1:** Baseline clinical, tumor-related, and treatment characteristics of the response-evaluable population.

Variable	Value
Number of patients	148
Age at diagnosis, median (range)	64.5 (35–92)
Sex	
- Male	59%
- Female	41%
Clinical stage	
- I	10 (6.8%)
- II	42 (28.4%)
- IIIA	8 (5.4%)
- IIIB	52 (35.1%)
- IIIC	13 (8.8%)
- IV	23 (15.5%)
Tumor size on baseline MRI	
- <3 cm	36 (24.3%)
- 3–4 cm	23 (15.0%)
- 4–6 cm	46 (31.4%)
- ≥6 cm	43 (29.3%)
Tumor distance from anal verge	
- 0–5 cm	42 (28.3%)
- 5–10 cm	61 (41.2%)
- >10 cm	45 (30.4%)
Neoadjuvant treatment strategy	
- 5 × 5 Gy	3 (2%)
- 5 × 5 Gy + chemotherapy (FOLFOX) (6–8 cycles)	81 (54.7%)
- 50 Gy + capecitabine	44 (29.7%)
- 50 Gy + capecitabine + chemotherapy (FOLFOX) (6–8 cycles)	17 (11.6%)
- Other regimens	3 (2%)

**Table 2:** Neoadjuvant treatment strategies according to baseline clinical stage (*n* = 148).

Clinical Stage	5 × 5 Gy	5 × 5 Gy + Chemotherapy (FOLFOX) (6–8 Cycles)	50 Gy + Capecitabine	50 Gy + Capecitabine + Chemotherapy (FOLFOX) (6–8 Cycles)	Other Regimens	Total
Stage I	0	0	7	0	0	10
Stage II	1	17	22	2	0	42
Stage IIIA	0	3	5	0	0	8
Stage IIIB	1	36	8	6	1	52
Stage IIIC	1	9	2	1	0	13
Stage IV	0	16	0	8	1	23
Total	3	81	44	17	3	148

**Table 3:** Clinical complete response (cCR) according to baseline clinical stage in the response-evaluable population (*n* = 148).

Stage	cCR Yes/Total	cCR (%)
I	6/10	60%
II	22/42	52%
IIIA	1/8	12.5%
IIIB	20/52	38%
IIIC	4/13	31%
IV	3/23	13%

**Table 4:** Clinical complete response (cCR) according to baseline tumor size assessed by magnetic resonance imaging (MRI) in the response-evaluable population.

Tumor Size (MRI)	cCR Yes/Total	cCR (%)
<3 cm	19/34	56%
3–4 cm	12/21	57%

**Table 5:** Implementation of watch-and-wait strategy according to baseline tumor size assessed by magnetic resonance imaging (MRI) in the response-evaluable population.

Tumor Size (MRI)	W&W Yes/Total	W&W (%)
<3 cm	15/34	44%
3–4 cm	10/21	48%
4–6 cm	4/44	9%

**Table 6:** Clinical complete response (cCR) according to tumor distance from the anal verge in the response-evaluable population.

Distance	cCR (%)
0–5 cm	47%
5–10 cm	27%
>10 cm	48%

## Data Availability

The data supporting the findings of this study are available from the corresponding author upon reasonable request. The data are not publicly available due to privacy and ethical restrictions, as they contain information that could compromise the confidentiality of research participants.
